# Supplementary material for: Iterative evolution of large-bodied hypercarnivory in canids benefits species but not clades
Source: Commun Biol. 2020 Aug 21;3:461. doi: 10.1038/s42003-020-01193-9 (PMC7442796; doi:10.1038/s42003-020-01193-9)
Supplement: Supplementary file 1 — Supplementary Information [file 42003_2020_1193_MOESM1_ESM.pdf]

Supplementary Information for

Iterative Evolution of Large-Bodied Hypercarnivory in Canids Benefits Species but Not Clades

Mairin Balisi *et al.*

Supplementary Figures

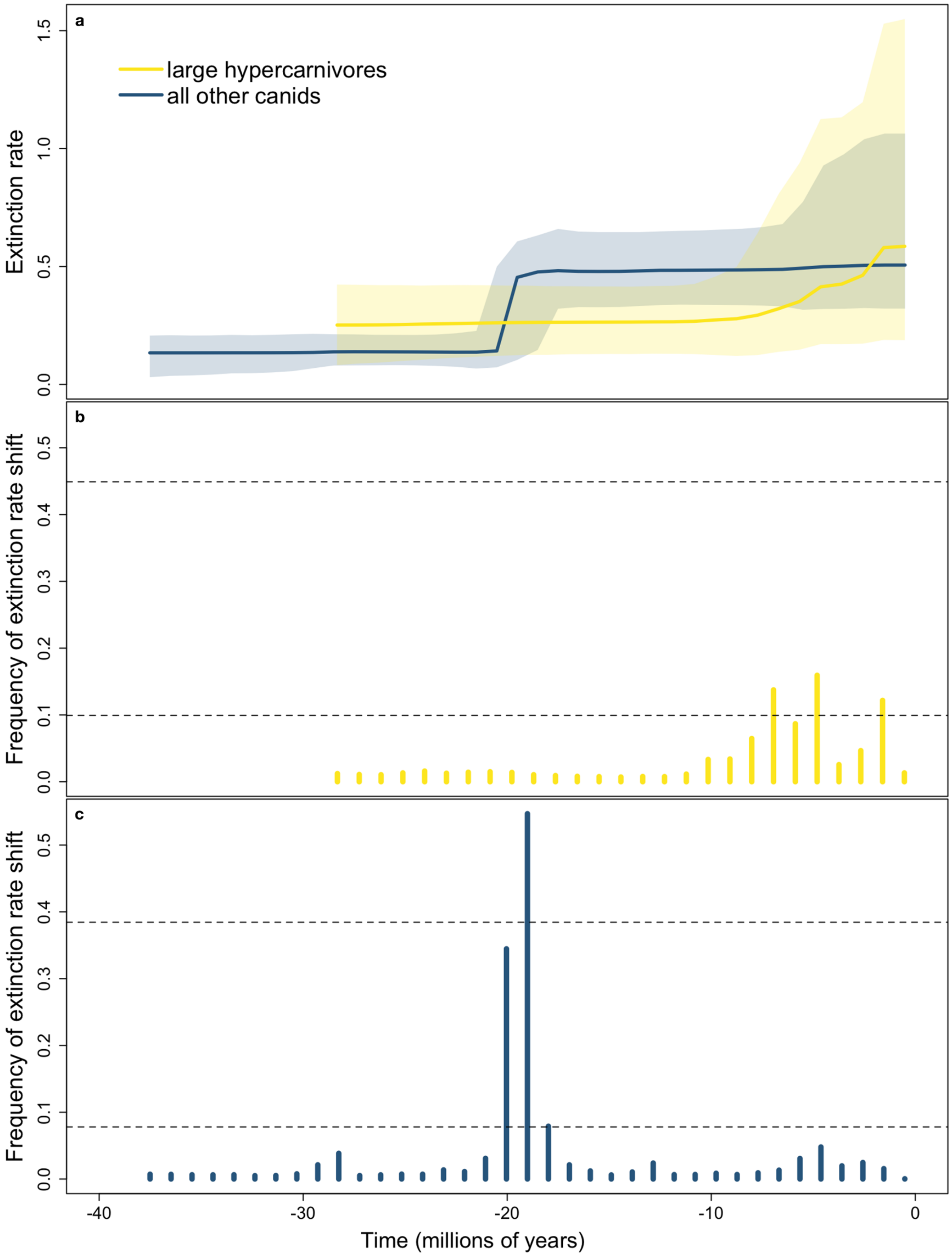

Supplementary Figure 1. a Extinction rates through time with 95% credible intervals based on 10,000,000 PyRate iterations, and **b, c** histograms of inferred times of rate shifts, for large hypercarnivores and all other canids. **a** is the same as Figure 3b in the Main Text. On the histograms, the horizontal dashed lines represent thresholds for positive evidence (bottom line) or strong evidence (upper line) of a rate shift, as determined by Bayes Factors (bottom line, logBF=2; upper line, logBF=6). Times of significant rate change—when significant posterior probability supports a rate shift—are indicated by bins in the histogram that show sampling frequencies for a rate shift exceeding the thresholds.

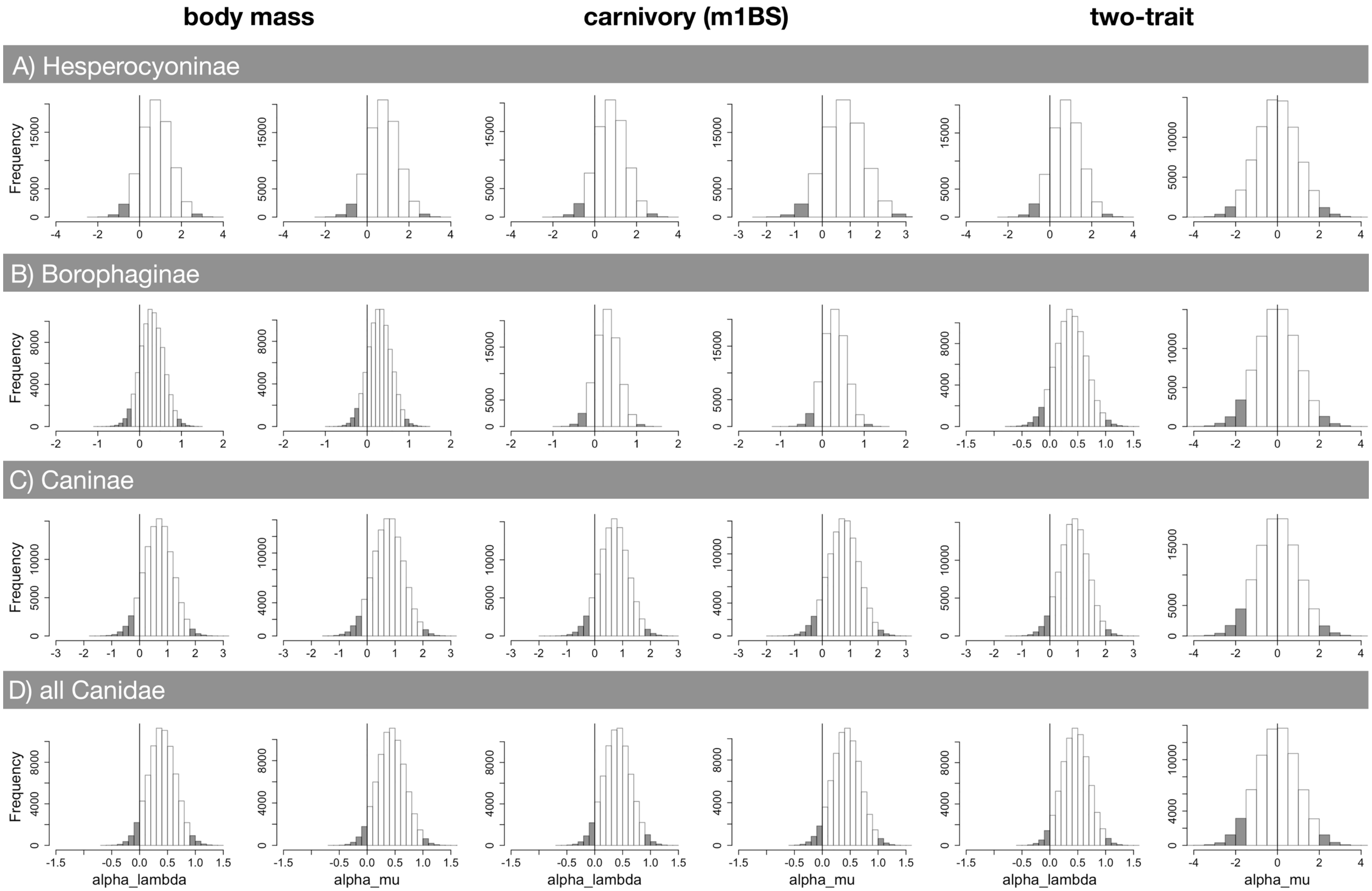

Supplementary Figure 2. Graphical representation of Supplementary Table 2: histograms illustrating weak (though insignificant) correlations between traits or trait combinations and some diversification rates. These include: two-trait ~ origination rate for Caninae, and all traits or trait combinations ~ all rates (except two-trait ~ extinction rate) for Canidae. alpha_lambda = correlation with origination rate; alpha_mu = correlation with extinction rate. If the 95% highest posterior density (white shading) spans 0, then the correlation is not considered significant.

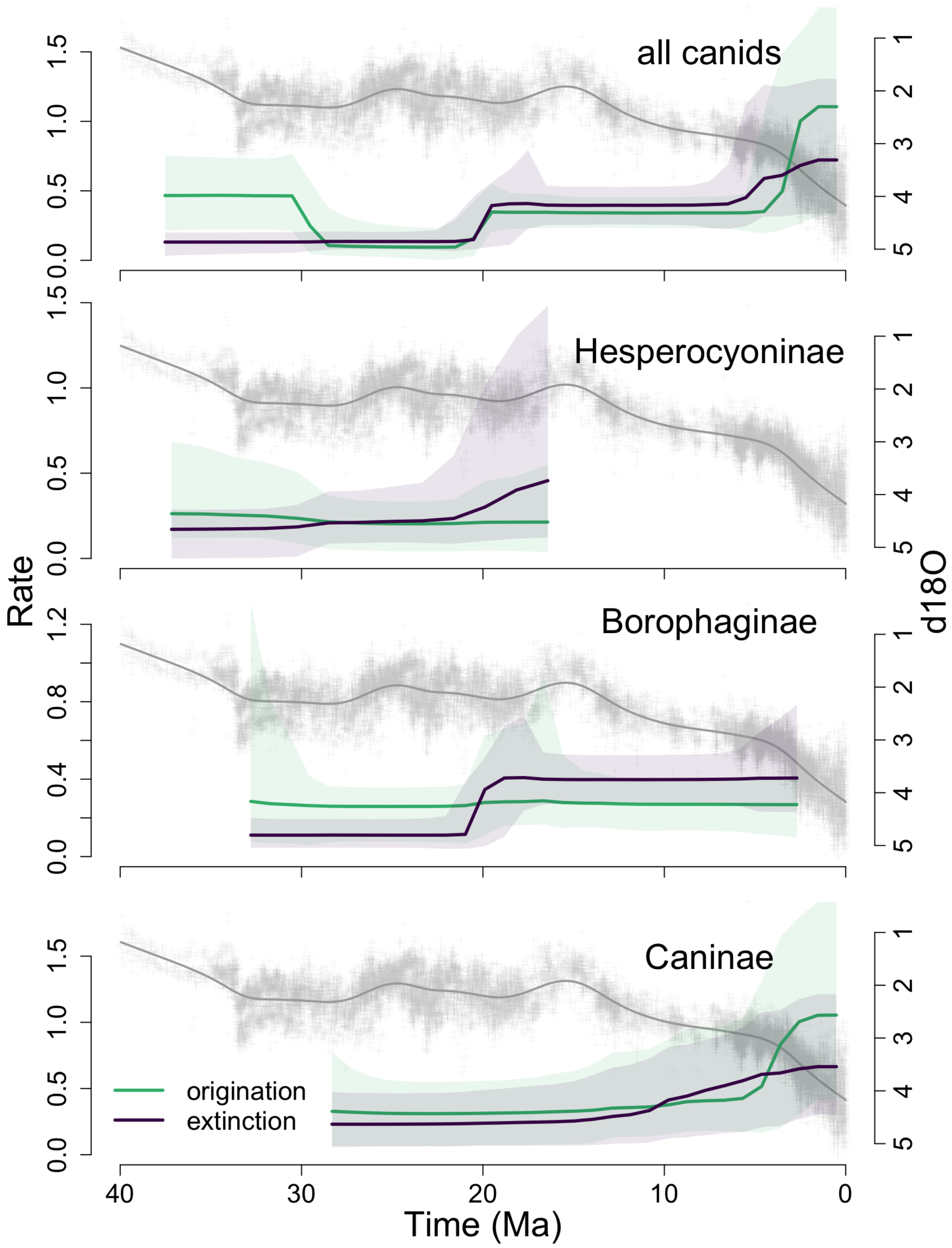

Supplementary Figure 3. Per-subfamily diversification rates of North American fossil canids superimposed on a smoothed temperature curve based on the Zachos *et al.* [1] global oxygen isotope record. Only all-Canidae and Caninae show correlation between diversification rates and global temperature; Hesperocyoninae and Borophaginae do not (Supplementary Table 3).

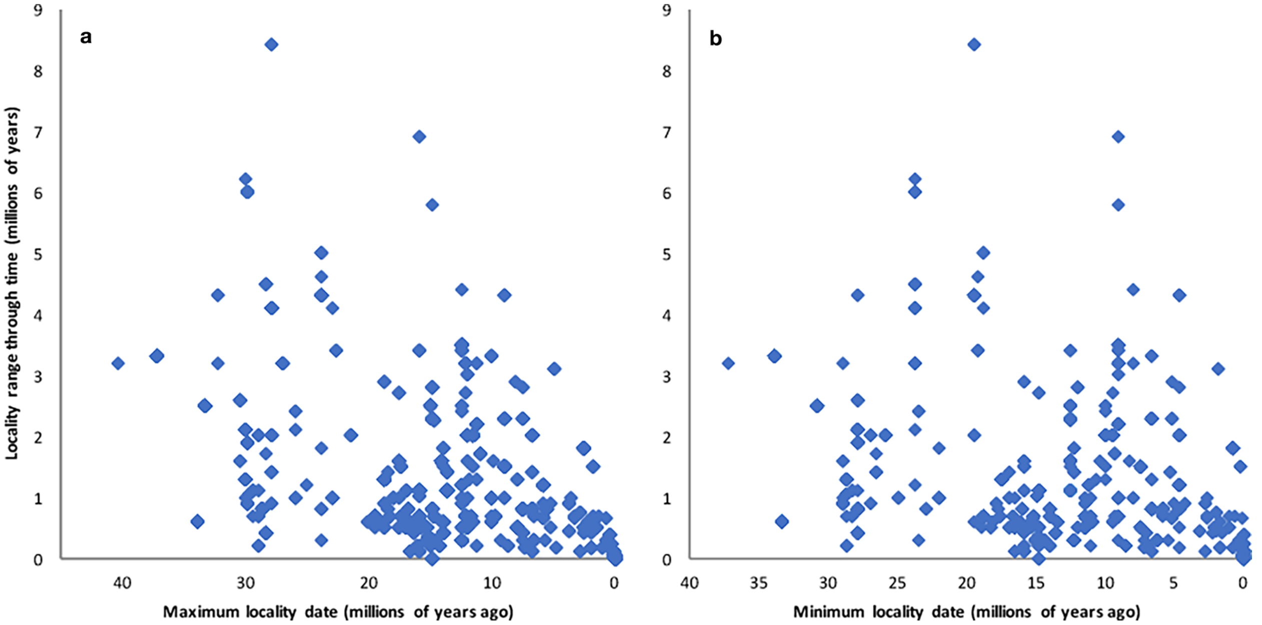

Supplementary Figure 4. Scatterplots of stratigraphic range through time against **a** maximum and **b** minimum date, with each point representing a single locality. Localities that are younger, whether judged by maximum or by minimum locality date, tend to have narrower time ranges (i.e. be more narrowly constrained by dating) than localities that are older. Maximum and minimum locality dates are plotted separately—rather than simply calculating average date per locality—because of this large variance in time range across localities and across time.

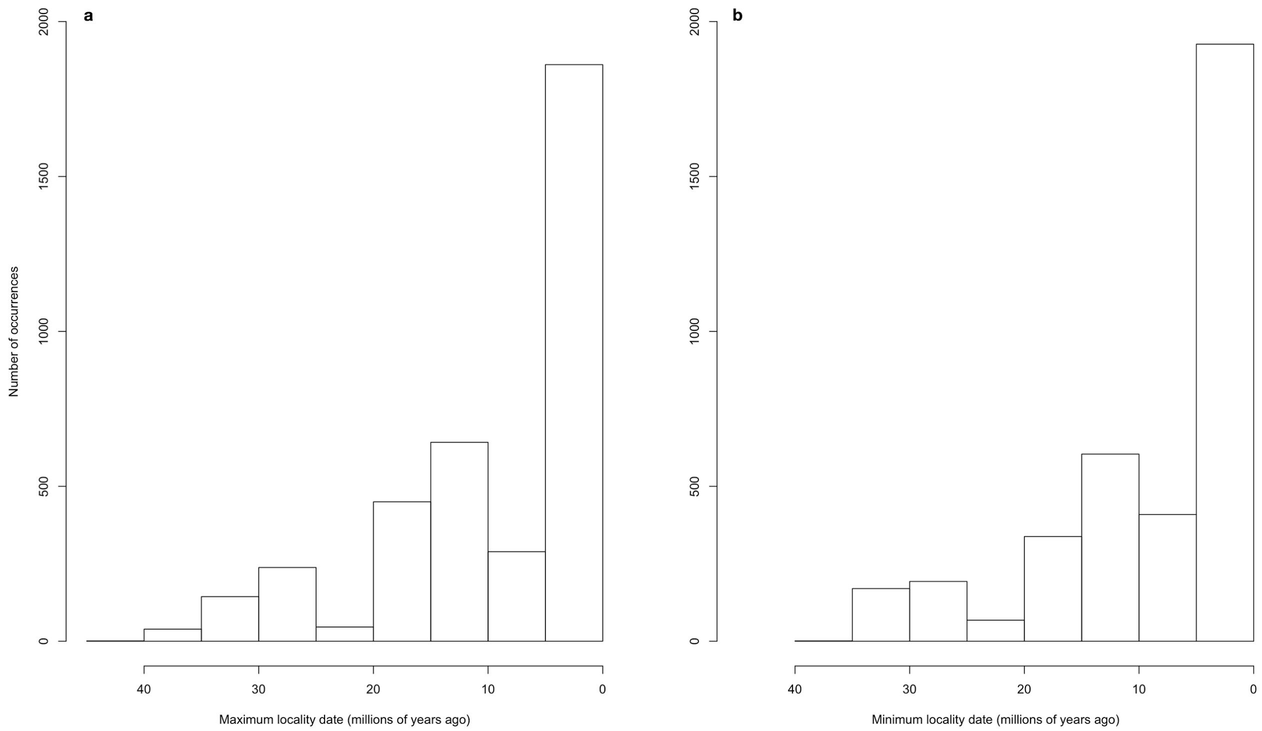

Supplementary Figure 5. Barplots of number of occurrences against **a** maximum and **b** minimum locality date. The number of occurrences ramps up toward the present. Maximum and minimum locality dates are plotted separately—rather than simply calculating average date per locality—because of the large variance in time range across localities and across time shown in Supplementary Figure 4.

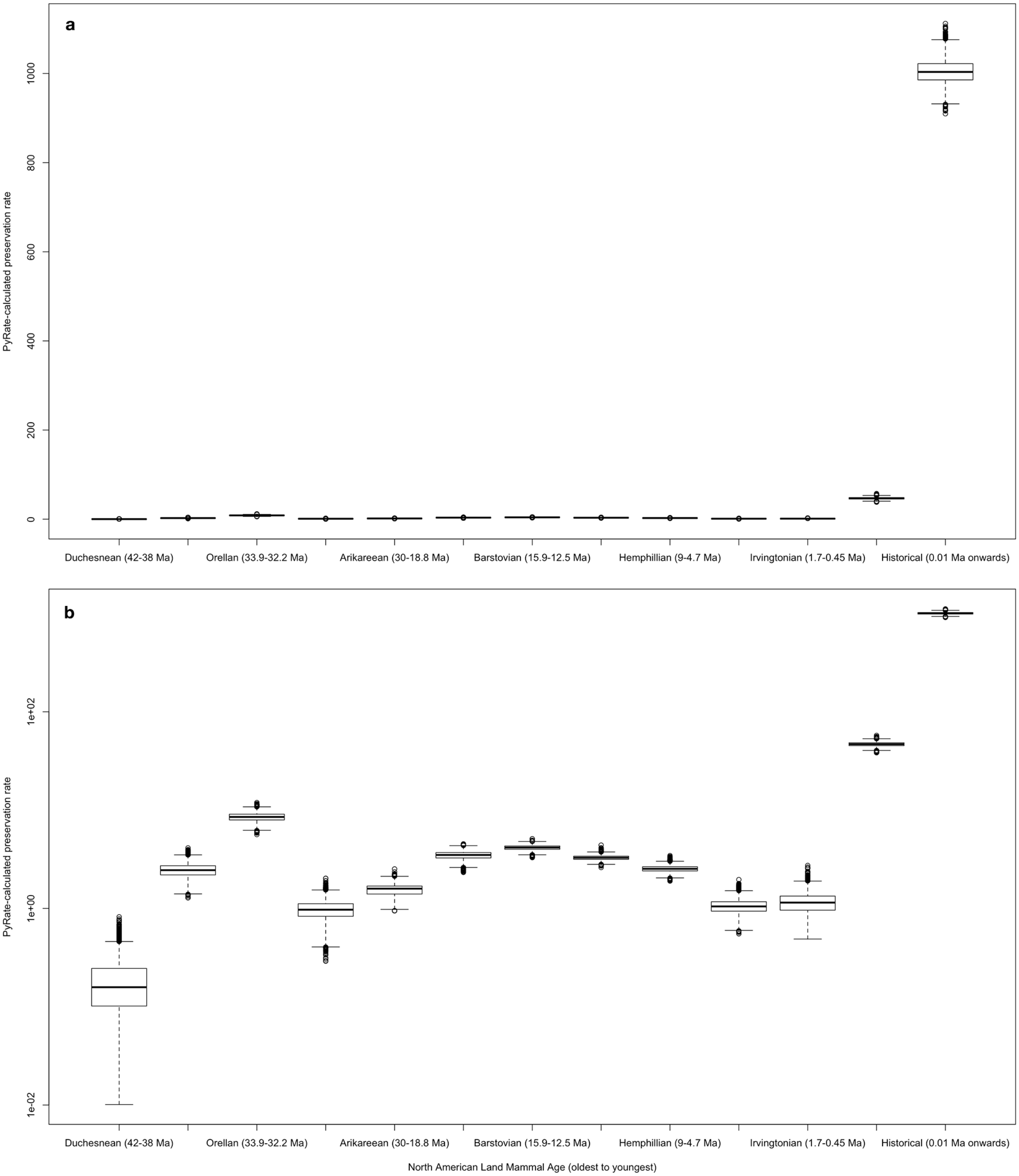

Supplementary Figure 6. Boxplots of preservation rate, calculated on 10,000,000 PyRate iterations, by North American Land Mammal Age (NALMA; Supplementary Table 4). **a** values plotted as-is; **b** has y-axis log-transformed because the discrepancy in preservation rate between the two most recent NALMAs and those preceding is too large, pushing all of the preceding preservation rates close to zero. Log-transformation highlights differences among these earlier time-bins, although preservation rates in these are still exponentially lower than the two most recent intervals.

Supplementary Tables

Supplementary Table 1. Relative probabilities of birth-death models with different numbers of rate shifts, reflecting differences in extinction rate patterns between large hypercarnivores and all other canids. Bolded numbers are the highest probabilities in each column, signifying the most probable model for origination or extinction in each category.

Model	LARGE HYPERCARNIVORES	ALL OTHER CANIDS
**1-rate**	0.2421	0
**2-rate**	**0.6163**	**0.6477**
**3-rate**	0.126	0.2787
**4-rate**	0.0144	0.0639
**5-rate**	0.0012	0.0088
**6-rate**	0.0001	0.0008
**7-rate**	0	0

Supplementary Table 2. Median posterior estimates of the parameters α_λ (correlation with origination rate) and α_μ (correlation with extinction rate) quantifying the relationship between traits and diversification rates. 95% highest posterior densities (HPD), based on 10,000,000 PyRate iterations, are in parentheses. Parameters displaying a significant relationship (95% HPD does not span 0) would be in bold, but no parameters were significantly correlated with diversification rates. However, because many parameters appear to be weakly correlated, these results also are presented graphically as histograms in Supplementary Figure 2.

	**Body mass**		**Carnivory (m1BS)**		**Two-trait**	
**Group**	α_λ	α_μ	α_λ	α_μ	α_λ	α_μ
Hesperocyoninae	0.7796 (-0.6376, 2.1522)	0.782 (-0.6075, 2.1987)	0.7808 (-0.6562, 2.1738)	0.7781 (-0.5773, 2.2365)	0.7809 (-0.5936, 2.1998)	-0.0104 (-2.0053, 1.9019)
Borophaginae	0.2969 (-0.2423, 0.8444)	0.2999 (-0.2392, 0.8526)	0.2982 (-0.2383, 0.8512)	0.2994 (-0.2481, 0.8407)	0.3745 (-0.1632, 0.9278)	-0.0009 (-1.9942, 1.9218)
Caninae	0.6727 (-0.3396, 1.6877)	0.7545 (-0.3473, 1.8331)	0.682 (-0.3584, 1.6756)	0.7545 (-0.34, 1.841)	0.8603 (-0.1323, 1.8755)	-0.0012 (-1.947, 1.9747)
All Canidae	0.4025 (-0.0917, 0.8721)	0.4356 (-0.0508, 0.9418)	0.4043 (-0.0891, 0.877)	0.4337 (-0.0564, 0.9451)	0.4615 (-0.0088, 0.9554)	2.828E-3 (-1.95, 1.9778)

Supplementary Table 3. Median posterior estimates of the parameters γ_λ and γ_μ quantifying the correlation between the Zachos *et al.* [1] global oxygen isotope record and diversification rates. 95% highest posterior densities (HPD), based on 1,050,000 PyRate iterations, are in parentheses. Parameters displaying significant correlation (95% HPD does not span 0) are in bold. NA's indicate inappropriate analyses, given that calculating origination rates for large hypercarnivores vs all other canids suggests that only large hypercarnivores can give rise to large hypercarnivores, which likely was not the case.

	**Exponential model**		**Linear model**	
**Group**	γ_λ	γ_μ	γ_λ	γ_μ
Large hypercarnivores	NA	0.816 (-0.2348, 2.8214)	NA	0.502 (-0.3272, 2.5199)
All other canids	NA	**1.9388 (0.8181, 2.8875)**	NA	**2.0708 (0.9871, 3.0486)**
Hesperocyoninae	0.0113 (-1.1218, 1.0209)	1.8046E-3 (-1.0593, 1.1385)	-0.0208 (-1.0405, 1.1069)	0.0314 (-1.3842, 1.0035)
Borophaginae	-0.4604 (-2.902, 0.6052)	0.4342 (-0.5575, 2.4346)	-0.3681 (-2.2691, 0.5688)	0.3488 (-0.5813, 1.821)
Caninae	**2.3019 (1.026, 3.4099)**	**1.9323 (0.7141, 3.3228)**	**2.5819 (1.3538, 3.7553)**	**2.6777 (0.9831, 3.9107)**
All Canidae	**2.2599 (1.4116, 3.2182)**	**2.3998 (1.4537, 3.1866)**	**1.4661 (0.8983, 1.9434)**	**2.1851 (1.3976, 2.866)**

Supplementary Table 4. Subdivisions of North American Land Mammal Ages (NALMA) used in this study. Intervals follow the age bounds in the MIOMAP / FAUNMAP database defined by Tedford *et al.* [2], except for the Early / Late Clarendonian pair of intervals, which were assigned to be of equal size after preliminary analysis showed that the original Early / Middle / Late Clarendonian subdivisions produced extreme unevenness in number of localities across the Clarendonian.

NALMA subdivision	Abbreviation	Lower bound (million years ago)	Upper bound (million years ago)
Orellan	OREL	33.7	32.2
Whitneyan	WHIT	32.2	30.0
Early Early Arikareean	EEAK (Ar1)	30.0	27.9
Late Early Arikareean	LEAK (Ar2)	27.9	23.8
Early Late Arikareean	ELAK (Ar3)	23.8	19.5
Late Late Arikareean	LLAK (Ar4)	19.5	18.8
Early Hemingfordian	EHMF (He1)	18.8	17.5
Late Hemingfordian	LHMF (He2)	17.5	15.9
Early Barstovian	EBAR (Ba1)	15.9	14.8
Late Barstovian	LBAR (Ba2)	14.8	12.5
Early Clarendonian	ECLA (Cl1/2)	12.5	10.75
Late Clarendonian	LCLA (Cl2/3)	10.75	9.0
Early Hemphillian	EHMP (Hh1/2)	9.0	6.7
Late Hemphillian	LHMP (Hh3/4)	6.7	4.7
Blancan	BLAN	4.7	1.7
Irvingtonian	IRVI	1.7	0.45
Rancholabrean	RANC	0.45	0.01
Holocene	HOLO	0.01	0

Supplementary References

1. Zachos, J. C., Dickens, G. R. & Zeebe, R. E. An early Cenozoic perspective on greenhouse warming and carbon-cycle dynamics. *Nature* **451**, 279–283 (2008).

2. Tedford, R. H. *et al.* Mammalian biochronology of the Arikareean through Hemphillian interval (late Oligocene through early Pliocene epochs). in *Late Cretaceous and Cenozoic Mammals of North America: Biostratigraphy and Geochronology* 169–231 (Columbia University Press, 2004).
