# Supplementary material for: Iterative evolution of large-bodied hypercarnivory in canids benefits species but not clades
Source: Commun Biol. 2020 Aug 21;3:461. doi: 10.1038/s42003-020-01193-9 (PMC7442796; doi:10.1038/s42003-020-01193-9)
Supplement: Supplementary file 2 — Reporting Summary [file 42003_2020_1193_MOESM2_ESM.pdf]

nature research

Corresponding author(s): Mairin Balisi

Last updated by author(s): Jul 7, 2020

Reporting Summary

Nature Research wishes to improve the reproducibility of the work that we publish. This form provides structure for consistency and transparency in reporting. For further information on Nature Research policies, see Authors & Referees and the Editorial Policy Checklist.

Statistics

For all statistical analyses, confirm that the following items are present in the figure legend, table legend, main text, or Methods section.

n/a	Confirmed	
☐	☒	The exact sample size (n) for each experimental group/condition, given as a discrete number and unit of measurement
☐	☒	A statement on whether measurements were taken from distinct samples or whether the same sample was measured repeatedly
☐	☒	The statistical test(s) used AND whether they are one- or two-sided<br>*Only common tests should be described solely by name; describe more complex techniques in the Methods section.*
☐	☒	A description of all covariates tested
☐	☒	A description of any assumptions or corrections, such as tests of normality and adjustment for multiple comparisons
☐	☒	A full description of the statistical parameters including central tendency (e.g. means) or other basic estimates (e.g. regression coefficient) AND variation (e.g. standard deviation) or associated estimates of uncertainty (e.g. confidence intervals)
☐	☒	For null hypothesis testing, the test statistic (e.g. F, t, r) with confidence intervals, effect sizes, degrees of freedom and P value noted<br>*Give P values as exact values whenever suitable.*
☐	☒	For Bayesian analysis, information on the choice of priors and Markov chain Monte Carlo settings
☐	☒	For hierarchical and complex designs, identification of the appropriate level for tests and full reporting of outcomes
☐	☒	Estimates of effect sizes (e.g. Cohen's d, Pearson's r), indicating how they were calculated

Our web collection on statistics for biologists contains articles on many of the points above.

Software and code

Policy information about availability of computer code

Data collection	No software was used for data collection.
Data analysis	We computed nonparametric test statistics using permutation (nonpartest() in R package npmv version 2.4.0) and adjusted for multiple comparisons using the Benjamini-Hochberg method (manually calculated using a spreadsheet). We quantified model support using Akaike weights (akaike.wts() in R package paleoTS version 0.5.2) based on Akaike Information Criterion values corrected for small sample sizes (AICc() in R package AICcmodavg version 2.2-2). The above procedures were executed in R version 3.6.1. Further, we used the open-source Python program PyRate v2.0 for Bayesian joint estimation of species richness, preservation rates, and diversification rates.

For manuscripts utilizing custom algorithms or software that are central to the research but not yet described in published literature, software must be made available to editors/reviewers. We strongly encourage code deposition in a community repository (e.g. GitHub). See the Nature Research guidelines for submitting code & software for further information.

Data

Policy information about availability of data

All manuscripts must include a data availability statement. This statement should provide the following information, where applicable:

- Accession codes, unique identifiers, or web links for publicly available datasets
- A list of figures that have associated raw data
- A description of any restrictions on data availability

The datasets generated and analyzed in the current study are available on the Dryad repository:
https://datadryad.org/stash/share/q29H2eayUbZ2ZN9jFl_TT7Px11vE9bNm4TN0H1RWL4k (link for reviewers)
https://doi.org/10.6071/M3M08P (permanent link)

Field-specific reporting

Please select the one below that is the best fit for your research. If you are not sure, read the appropriate sections before making your selection.

☐ Life sciences ☐ Behavioural & social sciences ☒ Ecological, evolutionary & environmental sciences

For a reference copy of the document with all sections, see nature.com/documents/nr-reporting-summary-flat.pdf

Ecological, evolutionary & environmental sciences study design

All studies must disclose on these points even when the disclosure is negative.

Study description	We estimated predator and prey body sizes and calculated a dental proxy for diet to categorize large hypercarnivores from all other canids; of 132 analyzed species, 99 were not large hypercarnivores. We quantified turnover over 17 time slices to pinpoint differences in body mass and diet between extinction survivors and victims. We compared diversification rates between large hypercarnivores and all other canids as well as among subfamilies. Lastly, we tracked diversification rates in correlation with (1) traits as potential intrinsic drivers and (2) global temperature estimated by oxygen isotopes as a possible extrinsic driver of canid diversification.
Research sample	All species of North American canids over the last 40 million years, from time of origin to the present.
Sampling strategy	No sample size predetermination was performed because (1) the question was about canids as a whole and (2) preservation of fragmentary fossils typically determines the number of specimens that can be sampled for a single species. The maximum number of accessible specimens was sampled for the maximum number of species.
Data collection	Linear morphometrics were collected using digital Mitutoyo calipers and recorded by Mairin Balisi. When specimens were not easily accessible, we compiled measurements from the literature. These measurements were correlated with occurrence data from the Neogene Mammal Mapping Portal (NEOMAP, http://ucmp.berkeley.edu/neomap) and Paleobiology Database (PBDB, http://www.paleobiodb.org). Oxygen isotope data approximating global temperature were obtained from Zachos et al. (2008, Nature).
Timing and spatial scale	Data were collected between 2013 and 2015. Frequency and periodicity of sampling are not relevant in this case because the samples are paleontological, already curated in public repositories, and unchanging in nature.
Data exclusions	No data were excluded from the analyses.
Reproducibility	Permutational statistics were run on 10,000 replicates, while Bayesian analyses using the open-access program PyRate were run for 10,000,000 iterations, reducing the probability of spurious conclusions.
Randomization	Sampled species were allocated into groups by phylogeny (three subfamilies of canids: Hesperocyoninae, Borophaginae, and Caninae), by body size (above and below 20 kg), by degree of carnivory (hypercarnivorous (eating over 70% meat) or not), and by extinction outcome (survived or extinct per time-slice).
Blinding	Blinding was attempted but not always possible because specimen labels bear species and genus names and because specimen size and morphology are often indicative of dietary ecology, which we investigate here.

Did the study involve field work? ☐ Yes ☒ No

Reporting for specific materials, systems and methods

We require information from authors about some types of materials, experimental systems and methods used in many studies. Here, indicate whether each material, system or method listed is relevant to your study. If you are not sure if a list item applies to your research, read the appropriate section before selecting a response.

Materials & experimental systems

n/a	Involved in the study	
☒	☐	Antibodies
☒	☐	Eukaryotic cell lines
☐	☒	Palaeontology
☒	☐	Animals and other organisms
☒	☐	Human research participants
☒	☐	Clinical data

Methods

n/a	Involved in the study	
☒	☐	ChIP-seq
☒	☐	Flow cytometry
☒	☐	MRI-based neuroimaging

Palaeontology

Specimen provenance	The study measured museum specimens; no new specimens were collected.

Specimen deposition	The measured specimens are in the collections of the American Museum of Natural History (New York, NY, US), University of California Museum of Paleontology (Berkeley, CA, US), Los Angeles County Natural History Museum (Los Angeles, CA, US), Yale Peabody Museum (New Haven, CT, US), and John Day Fossil Beds National Monument (Kimberly, OR).
Dating methods	No new dates are provided.

☐ Tick this box to confirm that the raw and calibrated dates are available in the paper or in Supplementary Information.
